# Transcriptional and epigenetic regulation of Ca^2+^-signaling genes in hepatitis B-derived hepatocellular carcinoma and their association with the cancer hallmarks

**DOI:** 10.1093/bioadv/vbaf331

**Published:** 2026-01-27

**Authors:** Guadalupe Hernández-Martínez, Andrés Hernández-Oliveras, Ángel Zarain-Herzberg, Juan Santiago-García

**Affiliations:** Programa de Doctorado en Ciencias de la Salud, Universidad Veracruzana, Xalapa, Veracruz 91190, México; Laboratorio de Biología Molecular, Instituto de Investigaciones Biológicas, Universidad Veracruzana, Xalapa, Veracruz 91190, México; Centro Científico y Tecnológico de Excelencia Ciencia & Vida, Fundación Ciencia & Vida, Santiago 8580702, Chile; Departamento de Bioquímica, Facultad de Medicina, Universidad Nacional Autónoma de México, CDMX 04360, México; Laboratorio de Biología Molecular, Instituto de Investigaciones Biológicas, Universidad Veracruzana, Xalapa, Veracruz 91190, México

## Abstract

**Motivation:**

Dysregulation of Ca^2+^-signaling genes has been shown in some types of cancer; however, it is virtually unknown in hepatitis B-derived hepatocellular carcinoma (HBV-HCC). Here, we evaluate the transcriptional and epigenetic regulation of Ca^2+^-signaling genes in HBV-HCC and whether their expression is associated with cancer hallmarks, and prognostic potential.

**Results:**

We identified 432 differentially expressed Ca^2+^-signaling genes in HBV-HCC, including 134 that are specific to this condition, and were not found in non-HBV HCC. Fifty-three of these genes were associated with cancer hallmarks, of which 17 exhibited potential prognostic value by Cox multivariate analyses. We also provide new evidence for epigenetic regulation by post-transcriptional histone modifications and DNA methylation at the promoter of some of these genes. Finally, using Least Absolute Shrinkage and Selection Operator (LASSO) regression, we identified a four-gene prognostic signature (*FBLN1*, *STC2*, *C1R*, and *F2RL2*) that robustly stratified patient outcomes. This study presents the first integrative transcriptomic and epigenetic analysis of Ca^2+^-signaling genes in HBV-HCC, introducing a novel four-gene signature with prognostic potential. These findings highlight the relevance of a dysregulation of a subset of Ca^2+^-signaling genes as a distinctive feature of HBV-HCC.

**Availability and implementation:**

All data generated or analyzed during this study are included in this article.

## 1 Introduction

Liver cancer is the third leading cause of cancer death worldwide (GLOBOCAN 2022). Hepatocellular carcinoma (HCC) is a multifactorial pathology whose risk factors are hepatitis B and C virus infections, alcoholism, diabetes, aflatoxins, non-alcoholic steatohepatitis, cirrhosis, autoimmune hepatitis, genetic factors such as mutations, and altered epigenetic mechanisms such as DNA methylation, histone post-translational modifications, and non-coding RNAs (Braghini *et al.* 2022, Lin *et al.* 2025).

We have recently shown that the expression of several Ca^2+^-signaling genes is altered in HCC, including 22 genes with clinical relevance. Moreover, the expression of some of these genes was modulated during liver regeneration and showed correlation with DNA methylation and/or histone marks (Hernández-Oliveras *et al.* 2021). Ca^2+^-signaling is involved in multiple cellular processes, such as cell cycle, proliferation, apoptosis, gene expression, and metabolism, among others (Lipskaia and Lompré 2004, Prevarskaya *et al.* 2014, Stewart *et al.* 2015, Monteith *et al.* 2017). Deregulation of Ca^2+^-signaling genes can lead to the onset of various pathologies, including cardiac, renal, neurodegenerative diseases, and cancer (Wu *et al.* 2021, Bastioli *et al.* 2024, Hernández-Oliveras and Zarain-Herzberg 2024).

In hepatitis B virus infections, the viral protein HBx can bind to Ca^2+^-signaling proteins, leading to hepatic inflammation. For example, interaction with ORAI1 channels contributes to extracellular Ca^2+^ influx into the cytoplasm (Yao *et al.* 2018, Kong *et al.* 2021). Besides, HBx activates caspase-3, which cleaves plasma membrane calcium pumps (PMCA), preventing Ca^2+^ efflux to the extracellular space and overloading the cytosol (Chami *et al.* 2003). Furthermore, HBx increases Ca^2+^ efflux from the ER to the cytoplasm by activating IP3R and increasing CD36 via the Src pathway. Also, HBX can potentiate Ca^2+^ influx into the mitochondria by interacting with VDAC3/MCU, as well as Ca^2+^ efflux through mNCX and mPTP (McClain *et al.* 2007, Cho *et al.* 2011, Yang and Bouchard 2012, Kong *et al.* 2021).

Among the molecular mechanisms that trigger carcinogenesis in HBV-infected liver are inflammation, activation of reactive oxygen species that promote genomic instability, and metabolic reprogramming (D’souza *et al.* 2020). Sustained high levels of cytosolic Ca^2+^ activate several mitogenic factors involved in the development and progression of inflammatory liver diseases (Katz *et al.* 2007, Oliva-Vilarnau *et al.* 2018). In addition, several genes involved in Ca^2+^-signaling are closely related to the initiation, promotion, and progression of hepatocellular carcinoma, and their expression might be regulated by epigenetic mechanisms such as DNA methylation and/or histone post-translational modifications (Ali *et al.* 2020, Hernández-Oliveras *et al.* 2021, Lai *et al.* 2022). However, very little is known about the role of epigenetic mechanisms, such as DNA methylation and HPM, that regulate the Ca^2+^-signaling genes in HBV-HCC. Therefore, this study aimed to explore the expression of Ca^2+^-signaling genes in HBV-HCC, determine whether epigenetic mechanisms are associated with their transcriptional regulation, and understand whether HBV may generate a different transcriptional and epigenetic landscape compared to uninfected HCC.

## 2 Methods

### 2.1 Analysis of bulk RNA-seq data

Two datasets were used: GSE124535, including RNA-seq data from 35 HBV-HCC and non-tumoral paired tissue samples (Jiang *et al.* 2019, Zeng *et al.* 2020), and GSE94660, corresponding to 21 HBV-HCC tumor tissues and 21 non-tumoral liver samples (Yoo *et al.* 2017). The datasets were obtained from the Gene Expression Omnibus repository (GEO) and exported to the Galaxy Europe platform (Afgan *et al.* 2018). The sequence quality, base content, sequence duplication, per-sequence GC content, and adapter content were evaluated using the FastQC (v 0.52) tool. Trimming of adapters was performed with Trim Galore! (v 0.4.2) tool. The reads were aligned to the GRCh38 reference genome using HISAT2 (v 2.1.0). FeatureCounts (v 2.0.1) was used to count RNA-seq reads from SAM/BAM files, and DESeq2 (v 1.44.0) was used for differential expression analysis in RStudio (v 4.4.1). For differential gene expression analysis, VST transformation was applied, followed by removeBatchEffect in the limma package (v 3.60.6), considering the dataset as a batch variable to eliminate systematic effects between the GSE124535 and GSE94660 datasets.

Heatmaps were generated with the Heatmap2 tool (v 3.2.0). A log2 FoldChange >1 and <−1, and an adjusted *P* value <.05 based on the Benjamini-Hochberg FDR correction, were considered statistically significant. The list of differentially expressed genes (DEG) is included in Table 1, available as supplementary data at *Bioinformatics Advances* online. The Ca^2+^-signaling genes were filtered from the DEG list based on the list published by Hörtenhuber *et al.* (2017).

A differential expression analysis was also performed, using HBV-positive HCC samples (datasets GSE124535 and GSE94660) compared to HBV-negative HCC (dataset GSE114564) (Son *et al.* 2022, Eun *et al.* 2022, Park *et al.* 2022, Kim *et al.* 2023) to demonstrate the specific gene expression profile in HBV-HCC.

### 2.2 Cancer hallmarks association analysis

Differentially expressed Ca^2+^-signaling genes were considered for cancer hallmark analysis using the msigdbr package (v 7.5.1) in RStudio (https://igordot.github.io/msigdbr/). The MsigDB database (https://www.gsea-msigdb.org/gsea/msigdb/) was used to analyze cancer hallmark pathways. Data were classified as upregulated and downregulated, as shown in Table 3, available as supplementary data at *Bioinformatics Advances* online. Gene Ontology (GO) enrichment analysis was performed using the clusterProfiler package (v 4.12.6) in RStudio. An analysis of signaling pathway enrichment was also performed using the KEGG database (Kanehisa *et al.* 2023) to identify biological pathways associated with the signature of prognostic genes.

### 2.3 Multivariate analysis and LASSO regression

To evaluate the association between clinical and molecular variables with overall survival, a multivariate Cox proportional hazards model was applied, reporting hazard ratios (HR) with their respective 95% confidence intervals and *P* values using the TCGA-LIHC and GSE14520 (Roessler *et al.* 2010) databases. To select the most relevant variables and avoid overfitting, LASSO penalized regression was used with the glmnet package (v.4.1.10), employing 10-fold cross-validation to determine the optimal penalty parameter (λ). The model’s discriminatory ability to predict mortality was evaluated by calculating the concordance index (C-index) with the RMS package (v 8.0.0) and time-dependent ROC curves using the timeROC package (v0.4).

### 2.4 ChIP-seq analysis

We analyzed ChIP-seq data of Ca^2+^-signaling genes using the datasets: (i) GSE113879 from liver biopsies obtained from HCC patients with chronic HBV infection and (ii) GSE112221 from liver biopsies obtained from HCC patients with no report of prior HBV infection; both datasets included adjacent liver paired samples (Flecken *et al.* 2019, Hlady *et al.* 2019). Euchromatin marks, such as H3K4me1, H3K4me3, and H3K27ac, as well as the heterochromatin marks H3K9me3 and H3K27me3, were visualized. Accession numbers were obtained from the SRA, and sequencing quality was evaluated with FastQC (v 0.52). Reads were trimmed with Trim Galore! (v 0.4.2) and aligned against the human hg38 reference genome with Bowtie2 (v 2.5.3). MACS2 callpeak (v 2.1.1.20160309.2) was used for peak calling and visualized with the Integrative Genomic Viewer (https://igv.org/) (Robinson *et al.* 2023). A transcriptional regulatory region corresponding to 2 kb upstream and 2 kb downstream of the transcription starting site (TSS) was considered (Table 5, available as supplementary data at *Bioinformatics Advances* online).

### 2.5 DNA methylation profiling by array analysis

For the DNA methylation analysis of the altered Ca^2+^-signaling genes, we utilized two datasets: GSE73003, which contains HCC, HBV-HCC, and adjacent liver samples (Yamada *et al.* 2016), and GSE113017, which includes HCC and adjacent liver tissue (Shimada *et al.* 2019). The datasets were analyzed using the GEO2R platform, and the differential expression analysis was performed using limma (https://www.ncbi.nlm.nih.gov/geo/geo2r/). We selected the nearest cg probe to the TSS of each gene. A *P* value <.05 was considered statistically significant. DNA methylation and its correlation with gene expression were also explored using the Shiny Methylation Analysis Resource Tool (SMART) database (http://www.bioinfo-zs.com/smartapp/), with samples from the TCGA-LIHC project (Li *et al.* 2019).

## 3 Results

### 3.1 The expression of Ca^2+^-signaling genes is altered in HBV-HCC

**Figure 1:**
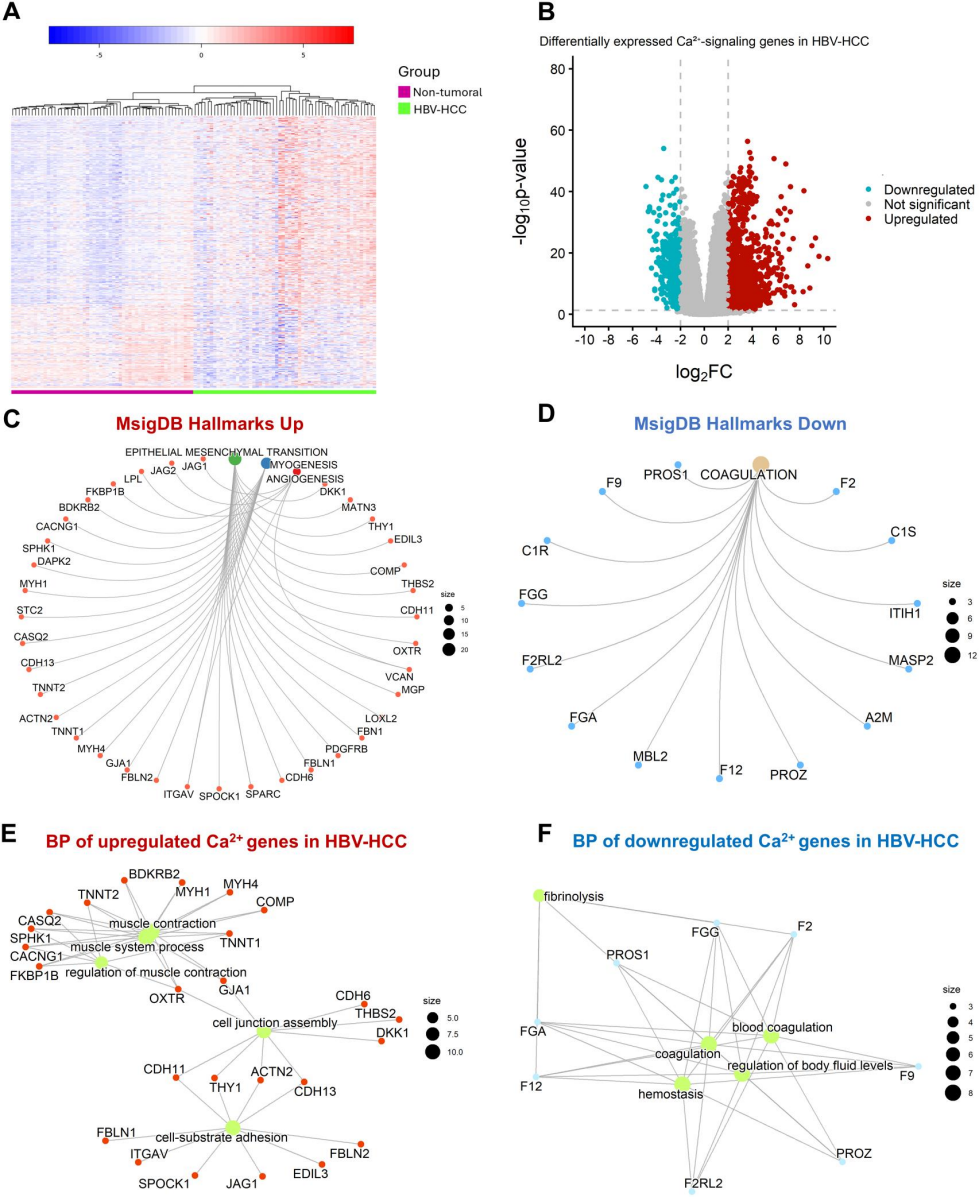
The expression of Ca^2+^-signaling genes is altered in HBV-HCC samples compared to adjacent liver tissue. Panel A shows a heatmap of upregulated and downregulated genes. Panel B shows a volcano plot of the 432 differentially expressed Ca^2+^-signaling genes. Panels C and D show the cnetplots of up- and down-regulated genes associated with the cancer hallmarks, respectively. Panels E and F show the interaction plots according to biological process (BP) for the up- and down-regulated genes.

A total of 112 samples were analyzed, of which 56 correspond to non-neoplastic adjacent liver tissue and 56 correspond to HBV-HCC samples. Among the 1670 Ca^2+^-signaling genes analyzed, 432 were differentially expressed in HBV-HCC compared to control samples (Fig. 1A; Table 1, available as supplementary data at *Bioinformatics Advances* online), 298 genes were upregulated, and 134 were downregulated. Their distribution is shown as a volcano plot (Fig. 1B). We then compared the expression of differentially expressed Ca^2+^-signaling genes in HBV-HCC samples with those of non-HBV HCC, as well as normal with non-HBV HCC. We identified 517 and 333 DEGs, respectively (Fig. 1A and B, available as supplementary data at *Bioinformatics Advances* online). Venn diagram intersection analysis showed that 134 DEGs are specific to the HBV-HCC condition, 342 were shared between HBV-HCC and non-HBV HCC, and 82 were shared among the three conditions analyzed (Fig. 1C, available as supplementary data at *Bioinformatics Advances* online). These results suggest that these 134 DEGs may be involved in particular mechanisms of carcinogenesis associated with HBV infection. The complete data are available in Table 2, available as supplementary data at *Bioinformatics Advances* online.

### 3.2 The expression of Ca^2+^-signaling genes is associated with cancer hallmarks

We performed an enrichment analysis using the differentially expressed Ca^2+^-signaling genes and the MsigDB data on cancer hallmarks to focus on genes with cancer relevance. The results showed that 39 upregulated genes were mainly enriched in three hallmarks. Twenty genes were associated with epithelial-mesenchymal transition (EMT), 14 with myogenesis, and 5 with angiogenesis (Fig. 1C). On the other hand, 14 downregulated Ca^2+^-signaling genes were enriched in blood coagulation (Fig. 1D). Furthermore, we performed a GO enrichment analysis for biological processes (BP) for the up- and down-regulated genes to understand the function of the genes associated with the cancer hallmarks. Overexpressed genes were mainly enriched in cell-substrate adhesion, cell junction assembly, and muscle contraction regulation (Fig. 1E). Meanwhile, the downregulated genes were enriched in blood coagulation and hemostasis regulation (Fig. 1F). The complete data can be found in Table 3, available as supplementary data at *Bioinformatics Advances* online.

### 3.3 Identification of a multi-gene prognostic signature in HBV-HCC

**Figure 2:**
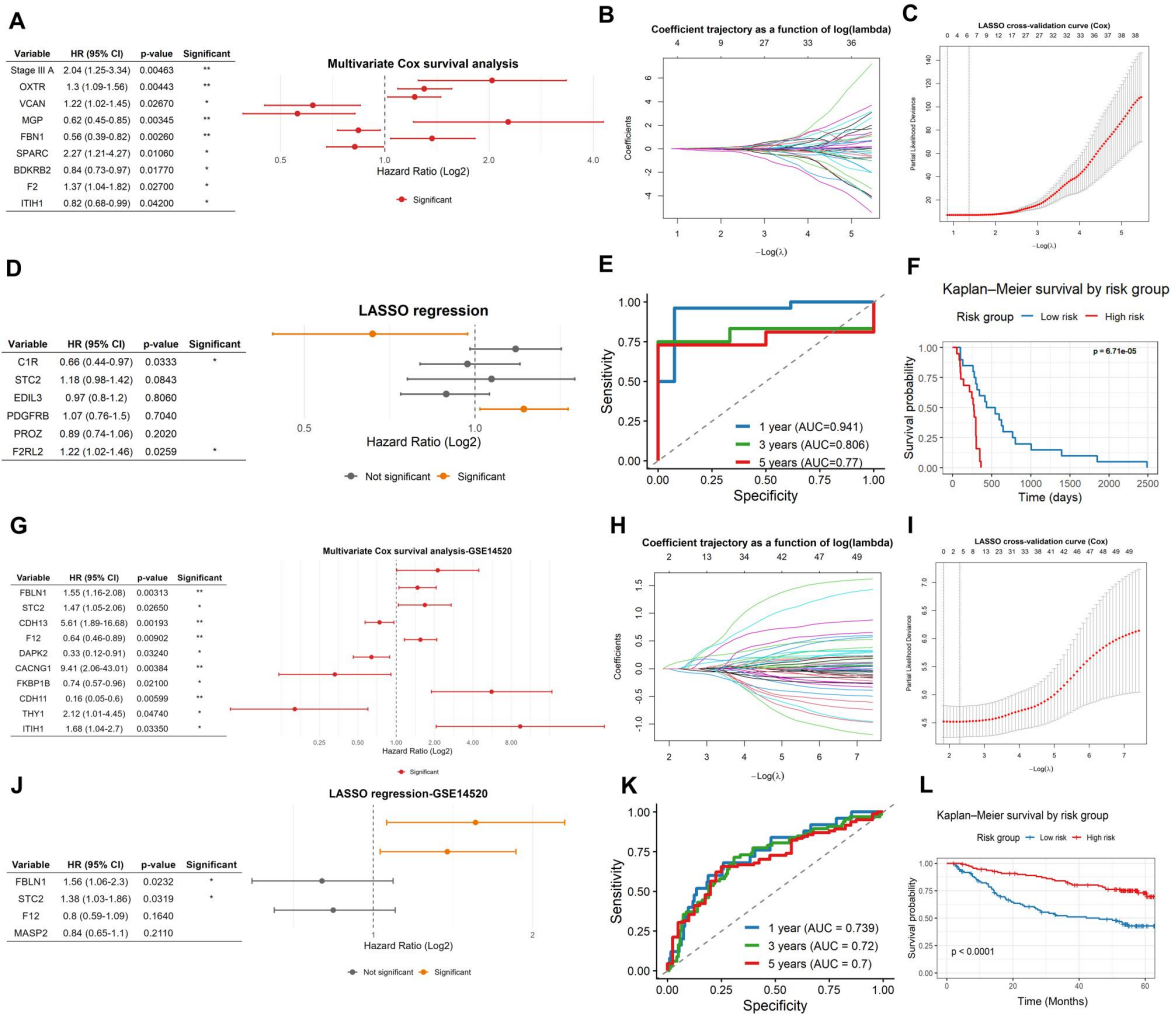
Identification and validation of a prognostic gene signature in HBV-HCC. (A) Forest plot showing a multivariate Cox regression analysis of Ca^2+^-signaling genes, using the TCGA-LIHC dataset. (B, C) Cross-validation selected six genes with non-zero coefficients at the optimal penalty (λ.min = 0.254). (D) Penalized LASSO regression reduced this set to a two-gene signature (*C1R* and *F2RL2*) with high predictive accuracy (C-index = 0.725). (E) The model showed excellent 1-year discrimination (AUC = 0.941); however, its accuracy declined at 3 and 5 years (AUC = 0.806 and 0.770, respectively), and significantly stratified patients into high- and low-risk groups (F). (G) Forest plot showing a multivariate Cox regression analysis of dataset GSE14520. (H, I) Cross-validation selected four genes with non-zero coefficients at the optimal penalty (λ.min = 0.1001). (J) LASSO regression identified a two-gene signature (*FBLN1* and *STC2*), with acceptable accuracy at 1 and 3 years (AUC = 0.739 and 0.72), and reduced performance at 5 years (AUC = 0.7) (K). Kaplan-Meier analysis confirmed effective risk stratification (L).

To evaluate the prognostic significance of Ca^2+^-signaling genes, we performed multivariate Cox regression models using cancer hallmarks-associated genes in two cohorts (TCGA-LIHC and GSE14520). In the TCGA-LIHC cohort, this analysis identified a set of genes with independent prognostic value (*OXTR*, *VCAN*, *MGP*, *FBN1*, *SPARC*, *BDKRB2*, *F2*, *ITIH1*) (Fig. 2A).

Penalized LASSO regression analysis was performed with the 53 DEGs associated with cancer hallmarks, and the optimal lambda penalty parameter was λ.min = 0.254, corresponding to the lowest validation error. At this point, six genes (*C1R*, *STC2*, *EDIL3*, *PROZ*, and *F2RL2*) with non-zero coefficients were selected, as shown in Fig. 2B and C.

After applying penalized LASSO regression, the model was reduced to a robust two-gene signature (*C1R* and *F2RL2*), with a C-index of 0.725 (Fig. 2D). In addition, the model demonstrated excellent discrimination at 1 year (AUC = 0.941), good discrimination at 3 years (AUC = 0.806), and moderate discrimination at 5 years (AUC = 0.770) (Fig. 2E), discriminating high-risk patients with poorer survival outcomes (Fig. 2F).

Validation in an independent cohort (GSE14520), confirmed the prognostic value of several genes (*FBLN1*, *STC2*, *CDH13*, *F12*, *DAPK2*, *CACNG1*, *FKBP1B*, *CDH11*, and *THY1*) (Fig. 2G). The validation curve showed that the penalty value reached its minimum value at λ.min = 0.1001 (–log λ ≈ 2.30). At this point, four genes (*FBLN1*, *STC2*, *F12*, and *MASP2*) with non-zero coefficients were selected (Fig. 2H and I). LASSO regression identified *FBLN1* and *SCT2* as the strongest predictors, with a C-index of 0.622 (Fig. 2J). In addition, the model demonstrates acceptable predictive accuracy at 1 year and 3 years (AUC = 0.739, 0.72); however, performance declines at 5 years (AUC = 0.7) (Fig. 2K). It also discriminates high-risk patients with poorer survival and those with low-risk (Fig. 2L). Overall, the multi-gene models demonstrated accurate prediction of early survival outcomes, while reduced long-term performance may reflect the influence of additional clinical factors. These findings support the potential of Ca^2+^-signaling genes as prognostic biomarkers in HCC, warranting further validation in prospective clinical settings.

### 3.4 HBV-HCC samples show different histone modification patterns at the promoter region of Ca^2+^-signaling genes compared to non-tumoral liver tissue

Differential histone post-translational modifications, such as methylation and acetylation, have been shown at the promoter regions of some Ca^2+^-signaling genes in HCC (Hernández-Oliveras *et al.* 2021). However, there is limited information for HBV-HCC. Therefore, we analyzed euchromatin-associated histone marks H3K4me1, H3K4me3, and H3K27ac, as well as heterochromatin marks H3K9me3 and H3K27me3 of Ca^2+^-signaling genes altered in HBV-HCC, obtained from a multivariate analysis, to assess whether these epigenetic mechanisms could explain changes in their expression levels. ChIP-seq datasets GSE113879 from HBV-HCC and GSE112221 from HCC samples were used to analyze a regulatory region spanning 2 kb upstream and 2 kb downstream of the transcriptional start site. The coordinates are provided in Table 4, available as supplementary data at *Bioinformatics Advances* online.

**Figure 3:**
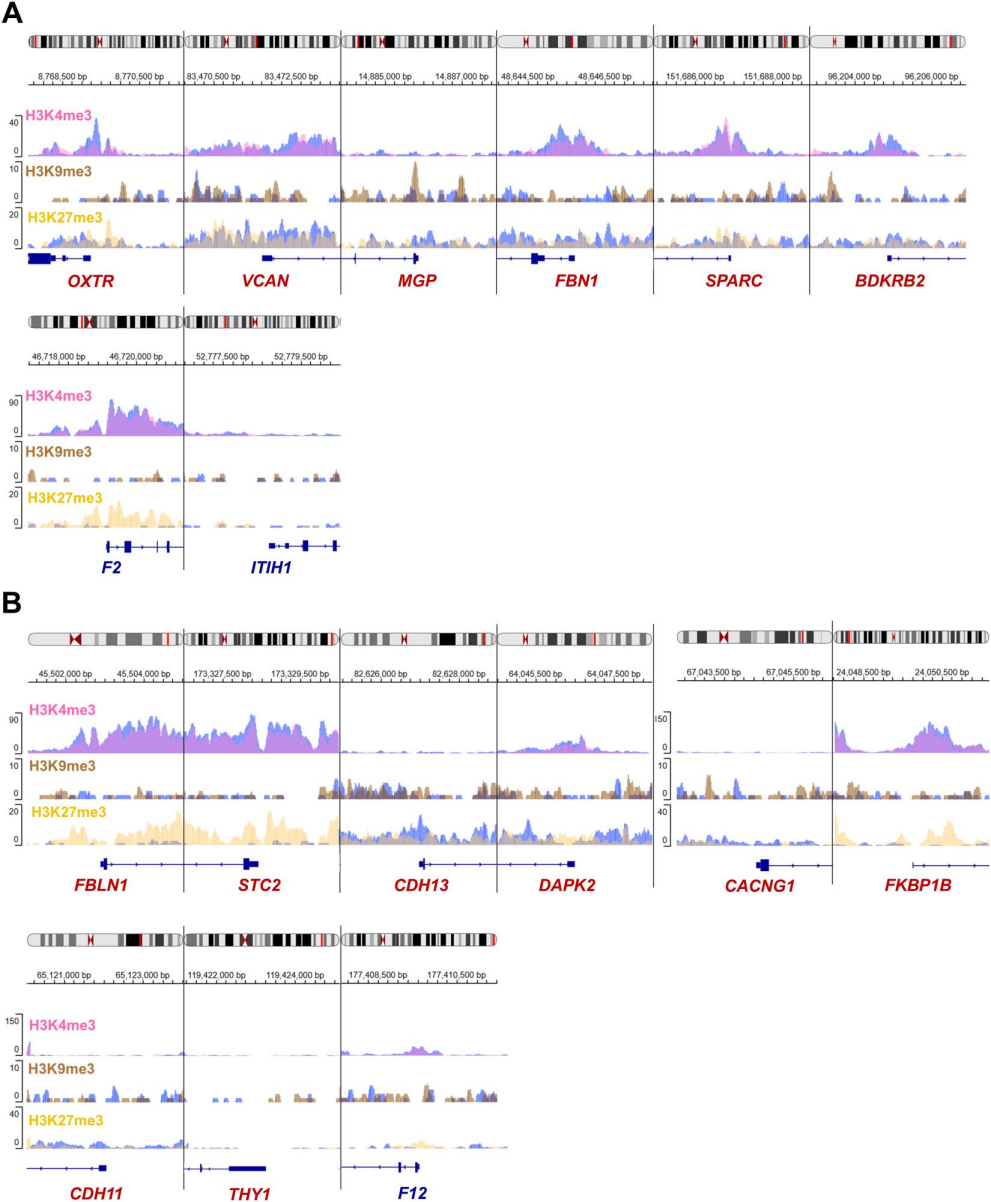
Post-translational modifications of the histone H3 at the promoter regions of differentially expressed genes in HBV-HCC (GSE73003). Panels A and B show the genes identified by multivariate analyses of TCGA-LIHC and GSE14520, respectively. The euchromatin-associated mark H3K4me3 is shown in lilac, whereas the heterochromatin marks H3K9me3 and H3K27me3 are represented in brown and yellow, respectively. Blue peaks correspond to histone modifications in control liver samples. The *x*-axis represents the genomic position relative to the TSS (−2000 bp to +2000 bp), and the *y*-axis indicates normalized ChIP-seq signal intensity. Blue arrows at the bottom indicate the gene orientation. The color of the gene symbols represents upregulated (red) or downregulated (blue).

**Figure 4:**
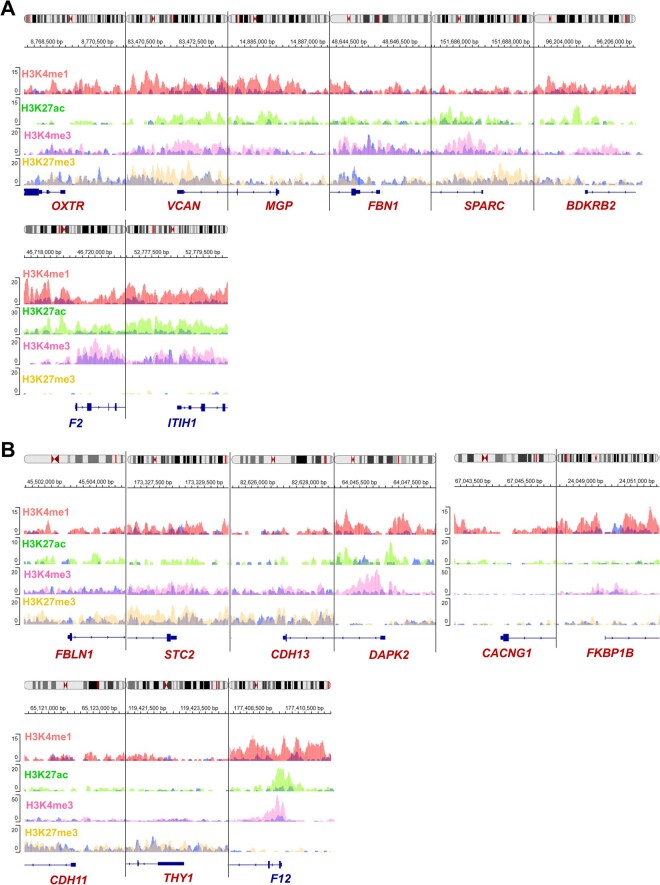
Post-translational modifications of the histone H3 at the promoter regions of differentially expressed genes in non-HBV HCC (GSE112221). Panels A and B show the genes identified by multivariate analyses of TCGA-LIHC and GSE14520, respectively. Three euchromatin-associated marks, H3K4me1 (red), H3K27ac (green), and H3K4me3 (lilac), are contrasted with the repressive heterochromatin mark H3K27me3 (yellow). Blue peaks represent signals from adjacent control liver samples. The *x*-axis represents the genomic position relative to the TSS as indicated by the gene symbol (−2000 to +2000 bp), and the *y*-axis indicates normalized ChIP-seq signal intensity. Blue arrows at the bottom denote the gene orientation. The color of the gene symbols represents upregulated (red) or downregulated (blue).

HBV-HCC patient samples show increased H3K9me3 at the promoters of *MGP* and *BDKRB2.* In addition, an increase in H3K27me3 was observed in the promoter of *F2* (Fig. 3A). In contrast, no changes in H3 marks were observed at the *OXTR*, *VCAN*, *FBN1*, *SPARC*, and *ITIH1* promoters (Fig. 3A). The promoters of *FBLN1*, *STC2*, and *FKBP1B* genes showed an increase in the H3K27me3 repression mark. However, the promoters of *CDH13*, *DAPK2*, *CACNG1*, *CDH11*, *THY1*, and *F12* genes showed no changes in histone marks (Fig. 3B). Subsequently, we analyzed the GSE11221 dataset, containing data for three activation marks (H3K4me1, H3K27ac, and H3K4me3) and the transcriptional repression mark H3K27me3, for non-HBV HCC and control liver samples. The results indicate increased H3K4me1 and H3K27ac marks at the promoters of *BDKRB2*, *F2*, and *ITIH1* in HCC. The *VCAN* and *SPARC* promoters showed an increase in H3K4me1, H3K27ac, and H3K27me3, whereas *OXTR* and *MGP* showed an increase in H3K4me1. Only the *FBN1* promoter showed no changes in histone marks (Fig. 4A).

On the other hand, the promoter of the downregulated gene *F12* showed increased levels of the three analyzed activation marks. The *FBLN1* and *STC2* promoters exhibited increased H3K4me1, H3K27ac, and H3K27me3 marks. The *CACNAG1*, *FKBP1B*, and *CDH11* promoters exhibited an increase in H3K4me1. The promoter of the *DAPK2* gene displayed upregulation of H3K4me1 and H2K27ac. However, the *CDH13* and *THY1* genes showed no changes in histone marks (Fig. 4B).

The ChIP-seq results suggest that histone post-translational modifications contribute to the transcriptional regulation of a subset of Ca^2+^-signaling genes in HBV-HCC. However, these epigenetic marks do not fully explain the up- or downregulation of all differentially expressed genes. This suggests that additional regulatory mechanisms, such as DNA methylation, chromatin accessibility, non-coding RNAs, and transcription factors binding, may also play important roles in the expression of the Ca^2+^-signaling genes in HBV-HCC and non-HBV HCC.

### 3.5 DNA methylation levels at the promoter of up- and downregulated Ca^2+^-signaling genes in HBV-HCC

We evaluated whether another epigenetic mechanism, such as DNA methylation, could explain the differential expression levels of Ca^2+^-signaling genes in HBV-HCC, with respect to the cancer hallmarks and prognostic relevance. We used datasets of DNA methylation profiles obtained from microarrays of non-tumor liver samples, non-HBV HCC, and HBV-HCC (GSE73003), and dataset GSE113017 from HCC samples.

**Figure 5:**
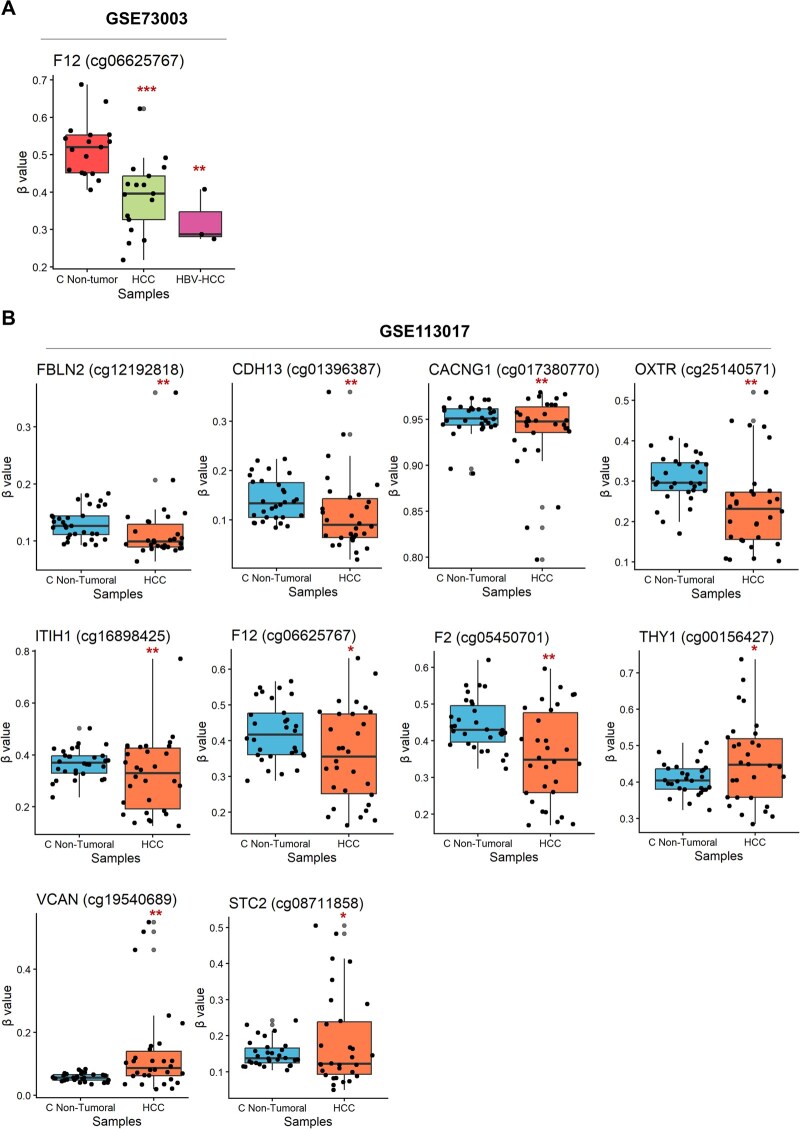
DNA methylation levels at the promoters of Ca^2+^-signaling genes in HBV-HCC and non-HBV HCC. Panel A shows DNA methylation level at the promoter of *F12* in non-HBV HCC and HBV-HCC samples, compared with adjacent control liver tissue. Panel B displays DNA methylation levels at the promoters of *FBLN1*, *CDH13*, *CACNG1*, *OXTR*, *ITIH1*, *F12*, *F2*, *THY1*, *VCAN*, and *STC2* in non-HBV HCC samples relative to adjacent tissue. DNA methylation was measured using CpG probes located within 200 bp upstream of the transcription start site. Statistical significance was determined using adjusted *P* values <.05.

The probes for each gene, near the transcription start site (200 bp), were used for the analysis. We found that, among all the genes analyzed, only the *F12* promoter (cg06625767) exhibited a decrease in promoter methylation in non-HBV HCC and HBV-HCC (GSE73003) (Fig. 5A). However, this does not explain the low expression of the gene.

In non-HBV HCC (GSE113017 dataset), the promoters of the *FBLN1* (cg12192818), *CDH13* (cg01396387), *CACNG1* (cg17380770), and *OXTR* (cg25140571) genes showed lower methylation levels than non-tumor samples, which would explain their high expression in non-HBV HCC (Fig. 5B).

In contrast, the promoters of the *ITIH1* (cg16898425), *F12* (cg06625767), and *F2* (cg05450701) genes showed lower methylation levels in non-HBV HCC than in control samples, which does not explain their downregulation. In addition, the promoters of *THY1* (cg00156427) and *VCAN* (cg19540689) showed higher methylation levels in non-HBV HCC than in control samples, which does not explain their upregulation (Fig. 5B).

Finally, the *STC2* promoter did not show a clear methylation pattern. The results suggest that changes in promoter methylation contribute to the transcriptional regulation of a subset of Ca^2+^-signaling genes in HBV-HCC and non-HBV HCC.

However, in most genes, differential promoter methylation does not explain their altered expression, indicating that other regulatory mechanisms may also be involved, such as enhancer methylation, repressor complexes (including HDACs, MBPs, MeCP2), methylation in non-promoter CpG islands, and non-coding RNAs.

We also investigated whether methylation levels correlate with gene expression in TCGA project samples, utilizing the SMART database. The results showed that the promoters of *F12* and *F2* are hypomethylated; however, the correlation between methylation and gene expression is negative, suggesting that their regulation in HCC does not depend on this epigenetic mechanism. In contrast, the *CDH13* promoter is hypermethylated and positively correlates with its expression, which does not explain its upregulation in HCC, suggesting the involvement of other regulatory mechanisms. Additionally, *CACNG1* exhibited a clear pattern, where promoter hypomethylation positively correlated with its overexpression, indicating that methylation may contribute to its transcriptional activation in HCC. For *FBLN1* and *OXTR*, no significant correlation between DNA methylation and gene expression was observed (Fig. 2, available as supplementary data at *Bioinformatics Advances* online). Overall, the results suggest that, except for *CACNG1*, DNA methylation does not appear to regulate the expression of Ca^2+^-signaling genes in non-HBV HCC and HBV-HCC.

Finally, we explored whether histone marks or DNA methylation regulated the expression of the prognostic gene signature obtained from LASSO regression. Histone marks from the GSE113879 and GSE112221 datasets for HBV-HCC and non-HBV HCC, respectively, were explored.

The results showed that histone marks do not directly associate with the expression of these genes (Fig. 3A and B, available as supplementary data at *Bioinformatics Advances* online). Furthermore, promoter methylation does not correlate with gene expression (Fig. 4, available as supplementary data at *Bioinformatics Advances* online). This suggests that these epigenetic mechanisms may not regulate the expression of these genes in HCC.

### 3.6 The prognostic signature of four genes in HBV-HCC contributes to key cellular processes relevant to cancer progression

We explored the signaling pathways in which these genes are involved using the KEGG database. *FBLN1* expression is important in cell adhesion processes (Fig. 5A, available as supplementary data at *Bioinformatics Advances* online), and *C1R* and *F2RL2* expression is important in coagulation (Fig. 5B, available as supplementary data at *Bioinformatics Advances* online). Even more importantly, in the context of cancer with epithelial-mesenchymal transition (*FBLN1*), coagulation (*C1R* and *F2RL2*), and myogenesis (*SCT2*), respectively (Fig. 1C and E). These results suggest that these genes may contribute to key cellular processes relevant to cancer progression, including adhesion, coagulation, and myogenesis.

## 4 Discussion

Approximately 1670 genes involved in Ca^2+^-signaling have been described so far (Hörtenhuber *et al.* 2017). However, the role of most Ca^2+^-signaling genes in HBV-HCC is not well understood. Based on high-throughput sequencing technology and bioinformatics analysis of two datasets containing paired HBV-HCC and adjacent control liver samples, we characterized the expression landscape of Ca^2+^-signaling genes in HBV-HCC. Gene expression analysis revealed that 432 Ca^2+^-signaling genes were altered in HBV-HCC, with 298 being upregulated and 134 downregulated. Among these genes, we explored those associated with cancer hallmarks and prognostic value. Among the upregulated genes, 20 correlated with the mesenchymal-epithelial transition, 14 with myogenesis, and 5 with angiogenesis, whereas the 14 downregulated genes were associated with coagulation.

It is important to note that we identified 134 specific Ca^2+^-signaling genes associated with HBV-HCC, which may be involved in specific carcinogenesis mechanisms related to viral infection. These findings support the hypothesis that dysregulation of Ca^2+^-regulated pathways may underlie HBV-specific mechanisms of hepatocarcinogenesis.

Previously, it has been described that the molecular alterations caused by HBV are very complex, as the oncoviral proteins HBx, HBsAg, HBeAg, and HBcAg can activate 10 cancer hallmarks: maintenance of proliferative signaling, evasion of tumor suppressor genes, evasion of immune destruction, inflammation, activation of invasion and metastasis, promotion of angiogenesis, genomic instability, resistance to cell death, replicative immortality, and metabolic reprogramming (D’souza *et al.* 2020, Sivasudhan *et al.* 2022). Our results suggest that Ca^2+^-signaling genes may play a relevant role in cancer hallmarks, particularly in HBV-HCC.

It has been reported that calcium channels or transporters, such as TRPM, STIM, and MUC, play a critical role in the EMT by upregulating mesenchymal markers, including Snail1, vimentin, N-cadherin, and twist, and downregulating the epithelial markers E-cadherin and claudin in some cancer types (Adiga *et al.* 2022). Calcium signaling also plays an important role in myogenesis, regulating myoblast proliferation and differentiation by recruiting transcription factors essential for muscle development. For example, Ca^2+^ influx from the endoplasmic reticulum to the cytosol via RyR3 activates calcineurin and triggers myogenic differentiation by activating MyoD and Mef2C (Sinha *et al.* 2022, Zhang *et al.* 2022).

Lung adenocarcinoma-derived CXCL1 is involved in myogenesis and alters the immune microenvironment of skeletal muscle, indicating a relationship between myogenesis and cancer (Hogan *et al.* 2018). Furthermore, increased myogenesis is associated with an enrichment of EMT and angiogenesis in gastric cancer (Chida *et al.* 2024). A close relationship between intracellular and endothelial Ca^2+^ oscillations has also been demonstrated, with cell migration, invasion, angiogenesis, and intravasation, mediated by growth factors such as vascular endothelial growth factor (VEGF), chemokines, and angiogenic modulators (Moccia 2020, Moccia *et al*. 2019, Adiga *et al.* 2022).

The liver synthesizes most coagulation factors and proteins involved in fibrinolysis. Calcium is essential for activating the coagulation and hemostasis pathways by activating thrombokinase with thromboplastin, which in turn converts prothrombin to thrombin. Our results showed downregulation of genes involved in coagulation, suggesting that coagulation may be altered in HBV-HCC (Millington-Burgess and Harper 2021).

In addition, calcium signaling genes differentially expressed in HBV-HCC and associated with cancer hallmarks (*OXTR*, *VCAN*, *MGP*, *FBN1*, *SPARC*, *BDKRB2*, *F2*, *ITIH1*, *FBLN1*, *STC2*, *CDH13*, *F12*, *DAPK2*, *CACNG1*, *FKBP1B*, *CDH11*, and *THY1*) showed prognostic potential according to the results of multivariate analysis with two independent HCC cohorts. The downregulated Ca^2+^-signaling genes in HBV-HCC correlated with the coagulation and complement systems. The role of HBV viral infections in coagulation, complement activation, inflammation, cytokine production, and activation of endothelial cells, leukocytes, and platelets has been previously elucidated (Liu *et al.* 2015, Zhang *et al.* 2021). This evidence suggests that while Ca^2+^ dysregulation is a general hallmark of HCC, HBV-related tumors exhibit distinctive patterns of extracellular matrix components and complement activation. Mechanistically, Ca^2+^-mediated signaling is well known to regulate EMT through cadherins and integrin signaling, and to promote angiogenesis via VEGF and *SPHK1* pathways (Moccia 2020, Adiga *et al.* 2022), supporting our hallmark-based classification.

In a different topic, it has been described that changes in epigenetic modifications, such as DNA methylation and HPM, can differentially regulate the transcription of several genes by altering the function of nucleosomes, which regulate chromatin compaction and, in turn gene expression (Muntean and Hess 2009).

Several Ca^2+^-signaling genes have been reported to be regulated by these mechanisms; for example, *ATP2A3* gene expression is regulated by histone acetylation in colon, gastric, breast, choroid plexus, lung, and liver cancer cells (Gélébart *et al.* 2002, Arbabian *et al.* 2013, Ait-Ghezali *et al.* 2014, Contreras-Leal *et al.* 2016, Flores-Peredo *et al.* 2017, Meneses-Morales *et al.* 2019, Hernández-Oliveras *et al.* 2019, 2021). Histone acetylation also regulates the *PMCA4* gene in breast cancer and *CDH1* in pancreatic cancer (Aghdassi *et al.* 2012, Varga *et al.* 2014). In addition, DNA methylation regulates *S100A4* and *S100P* genes in colon cancer, *DSC3* and *TGM2* in breast cancer, and *TGM2* in gastric cancer and glioma, respectively (Oshiro *et al.* 2005, Dyer *et al.* 2011, Chiang *et al.* 2015, Mudduluru *et al.* 2017, Kim *et al.* 2019). Histone post-translational modifications and DNA methylation are also deregulated in liver diseases such as alcoholic hepatitis, non-alcoholic fatty liver disease, and fibrosis (Mandrekar 2011, Page and Mann 2015, del Campo *et al.* 2018).

However, in HBV-HCC, the epigenetic mechanisms regulating the expression of Ca^2+^-signaling genes have not been described. Our results showed an increase in H3K9me3 at *MGP* and *BDKRB2* promoters, which do not explain their overexpression. In contrast, the *F2* promoter showed increased H3K27me3, which would explain its downregulation. However, no association was found between the H3K27me3 mark in the promoters of *FBLN1*, *STC2*, and *FKBP1B* and their corresponding expression levels. Furthermore, no changes in H3 marks were observed in the promoters of *OXTR*, *VCAN*, *FBN1*, *SPARC*, *ITIH1*, *CDH13*, *DAPK2*, *CACNG1*, *CDH11*, *THY1*, and *F12*, suggesting a different regulatory mechanism in HBV-HCC. Regarding non-HBV HCC, the upregulated genes *OXTR*, *VCAN*, *MGP*, *FBN1*, *SPARC*, *BDKRB2, FBLN1, STC2, DAPK2, CACNG1, FRBK1B*, and *CDH11* exhibited increased transcriptional activation mark H3K4me1. In addition, *VCAN*, *SPARC*, *BDKRB2*, *FBLN1*, *STC2*, and *DAPK2* showed an increase in H3K27ac, suggesting that these two transcriptional activation marks are relevant for the regulation of Ca^2+^-signaling genes in non-HBV HCC. However, the promoters of *VCAN*, *SPARC*, *FBLN1*, and *STC2* showed an increase in the repressive mark H3K27me3, which is discordant with their respective expression levels. Additionally, histone marks do not appear to regulate the expression of *F2*, *ITIH1*, and *F12*. These results provide new evidence of the potential role of HPMs in the transcriptional regulation of some differentially expressed Ca^2+^-signaling genes in HBV-HCC and non-HBV HCC.

On the other hand, DNA methylation did not show potential as a regulator of Ca^2+^-signaling genes in HBV-HCC. However, in non-HBV HCC, the promoters of the *FBLN1*, *CDH13*, *CACNG1*, and *OXTR* genes exhibited lower methylation levels than non-tumor samples, which would explain their high expression in HCC.

So far, the role of DNA methylation has been investigated in some Ca^2+^-signaling genes that are involved in coagulation and cell adhesion. For example, the *SPHK1* promoter is hypermethylated in highly differentiated HCC compared to poorly differentiated HCC. In JHH-7 and HLF cell lines, treatment with 5-Aza reduced DNA methylation levels and induced *SPHK1* expression (Tsuda *et al.* 2023). It has also been demonstrated that human papillomavirus integration affects *PROS1* gene expression in cervical cancer, and its low expression correlated negatively with DNA methylation levels (Zeng *et al.* 2023). DNA methylation in *C1R* was considered a prognostic biomarker for acute myeloid leukemia (Božić *et al.* 2015). Downregulation of *C1R* by hypermethylation led to increased malignancy in HCC cells through HIF-1α-mediated activation of glycolysis (Ma *et al.* 2024). Methylome and transcriptome analyses of liver biopsies revealed hypermethylation and downregulation of *C1R* and *C1S* in a model of metabolic dysfunction-associated steatohepatitis (Magdy *et al.* 2024).

Our findings on regulation by epigenetic mechanisms should be interpreted with caution, as the relationship between histone modifications and/or DNA methylation and gene expression is not strictly linear (Ptashne 2013). Activating or repressive histone marks do not act in isolation; they can coexist in the same promoter (bivalent domains), and their effects depend on the cellular context and cofactors (Bernstein *et al.* 2006, Esteller 2008). Furthermore, epigenetic regulation involves the dynamic interaction of distal enhancers, chromatin remodeling, transcription factors, non-coding RNAs (lncRNA, miRNA), 3D genome organization, as well as epigenetic plasticity (tumor heterogeneity) in cancer (Bernstein *et al.* 2006, Li *et al.* 2007, Esteller 2008, Ptashne 2013, Cavalli and Herad 2019, Nishiyama and Nakanishi 2021), which can lead to inconsistent results. Therefore, our data suggest an association of epigenetic marks and the expression of some genes, but do not allow us to conclude causality and require validation in future studies.

In addition, our results demonstrate the prognostic value of Ca^2+^-signaling genes in HCC, as reflected by the identification of multi-gene signatures with robust predictive performance in two independent cohorts. In the TCGA-LIHC, the two-gene signature (*C1R* and *F2RL2*) achieved excellent predictive accuracy, while in the GSE14520 cohort, *FBLN1* and *SCT2* emerged as the strongest predictors. Although the models showed high discrimination at 1–3 years, their performance declined at 5 years, suggesting that additional clinical and molecular factors beyond the analyzed gene sets influence long-term survival in HCC. These findings are consistent with previous studies linking Ca^2+^-signaling to tumor progression, angiogenesis, and metastasis, highlighting its mechanistic relevance in liver cancer biology (Prevarskaya *et al.* 2014, Monteith *et al.* 2017). However, the different genes selected in the two independent cohorts highlight the complexity of HCC and the need for validation to establish their usefulness as reliable prognostic biomarkers in prospective studies.

In conclusion, we identify 53 Ca^2+^-signaling genes with altered expression in HBV-HCC that are associated with the cancer hallmarks: EMT, angiogenesis, and myogenesis, as well as blood coagulation. Furthermore, our analysis provides new evidence for epigenetic regulation by post-translational histone modifications and DNA methylation of some of these genes, underscoring the importance of characterizing the transcriptional and epigenetic regulation of Ca^2+^-signaling genes in HCC to identify early markers of liver damage.

In addition, the four-gene prognostic signature obtained in our study (*C1R*, *F2RL2*, *FBLN1*, and *SCT2*) identified key processes relevant to HCC pathogenesis, including complement activation, coagulation, and extracellular matrix remodeling. Unlike larger multi-gene panels, this compact signature has the potential for translational value in clinical implementation. However, its predictive decline at 5 years suggests that integrating clinical variables and additional molecular features is necessary to achieve better prognostic performance.

## Supplementary Material

vbaf331_Supplementary_Data

## Data Availability

RNA-Seq datasets of HBV-HCC and non-HBV HCC used are available from Gene Expression Omnibus (GEO) under accession numbers GSE124535 (Jiang *et al.* 2019, Zeng *et al.* 2020), GSE94660 (Yoo *et al.* 2017), and HBV-negative HCC GSE114564 (Son *et al.* 2022, Eun *et al.* 2022, Park *et al.* 2022, Kim *et al.* 2023). Accession numbers for ChIP-Seq datasets of HBV-HCC and non-HBV HCC are GSE113879 and GSE112221 (Flecken *et al.* 2019, Hlady *et al.* 2019), whereas HBV-HCC and non-HBV HCC Methylation Array datasets used were GSE73003 (Yamada *et al.* 2016), and GSE113017 (Shimada *et al.* 2019).
