# Supplementary material for: Transcriptional and epigenetic regulation of Ca2+-signaling genes in hepatitis B-derived hepatocellular carcinoma and their association with the cancer hallmarks
Source: Bioinform Adv. 2026 Jan 27;6(1):vbaf331. doi: 10.1093/bioadv/vbaf331 (PMC12866915; doi:10.1093/bioadv/vbaf331)
Supplement: vbaf331_Supplementary_Data [file vbaf331_supplementary_data.zip › Supplementary_figures_BIOADV-2025-202.docx]

**Supplementary figures**


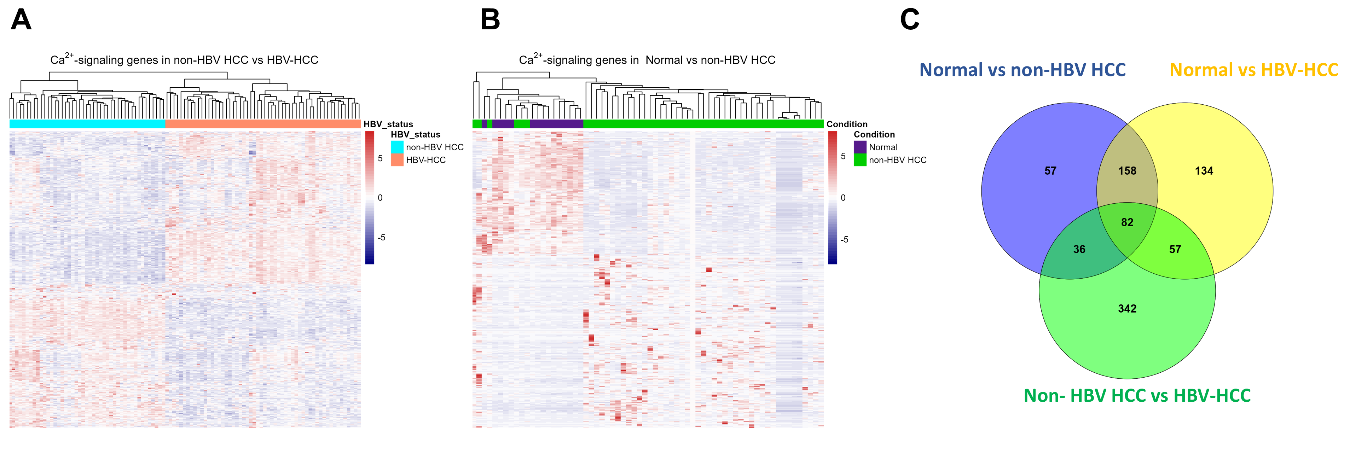


**Supplementary Figure 1.** Differential expression of Ca^2+^-signaling genes in HBV-HCC compared to normal tissue and non-HBV HCC. Panel A shows the 517 DEGs in non-HBV HCC compared to HBV-HCC. Panel B shows the 333 DEGs in non-HBV HCC compared to normal samples. The heatmaps represent upregulated (red) and downregulated (blue) genes. Panel C shows a Venn diagram obtained from the comparison between normal with non-HBV HCC (blue), normal tissue with HBV-HCC (yellow), and non-HBV HCC with HBV-HCC (green).


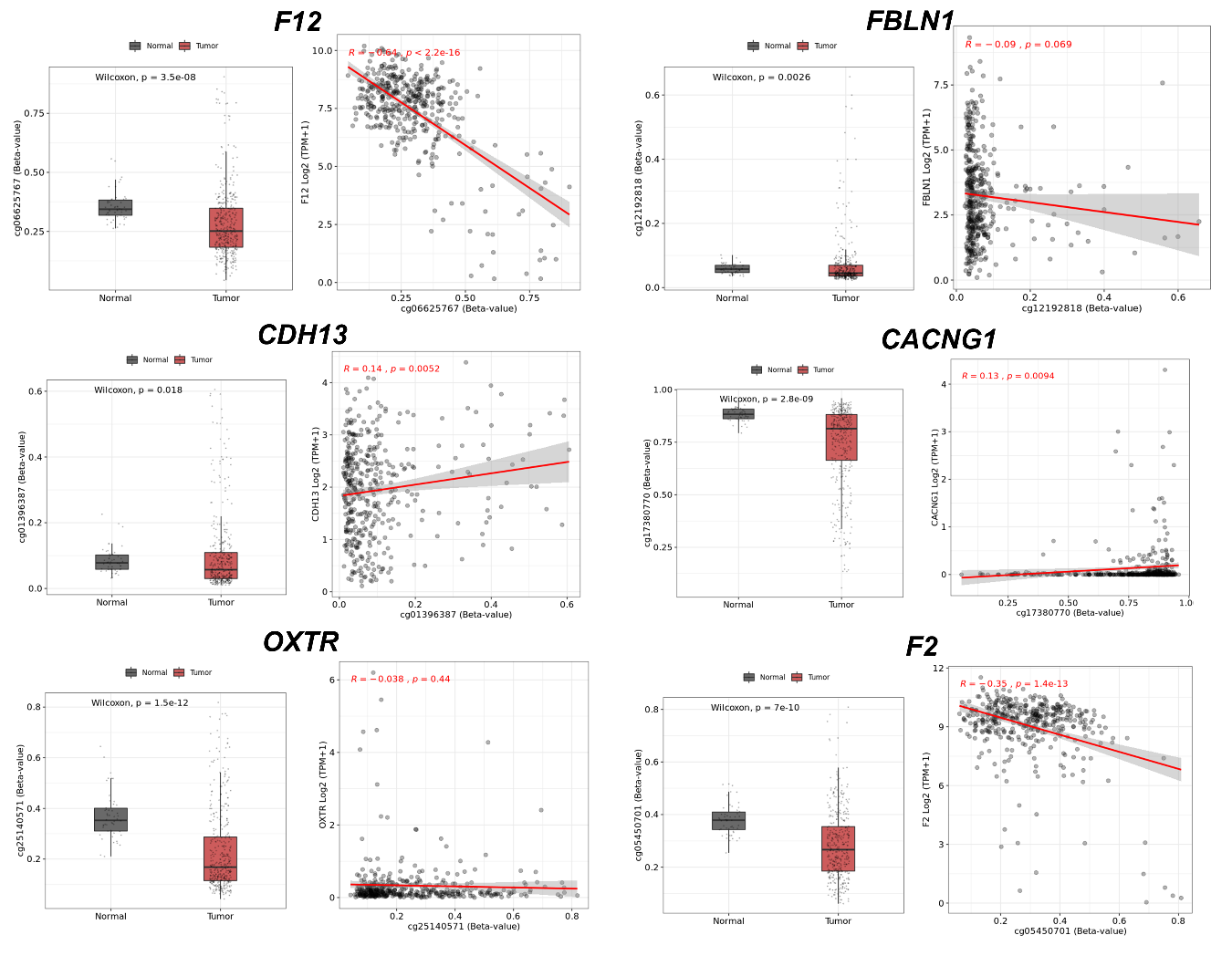


**Supplementary Figure 2.** Changes in DNA methylation at the promoters of Ca^2+^-signaling genes and correlation with gene expression, using TCGA-LIHC data. The figure shows DNA methylation levels at the promoters of *F12, FBLN1, CDH13, CACNG1, OXTR*, and *F2* in HCC samples (red) compared to adjacent control liver tissue (gray). DNA methylation was measured using CpG probes located 200 bp upstream of the transcription start site. The y-axis indicates β values representing the proportion of methylation (0 = unmethylated, 1 = fully methylated), while the x-axis corresponds to gene promoters. Additionally, correlation graphs of DNA methylation with each gene´s expression are shown, based on Pearson's correlation. The SMART database was used for the analysis.


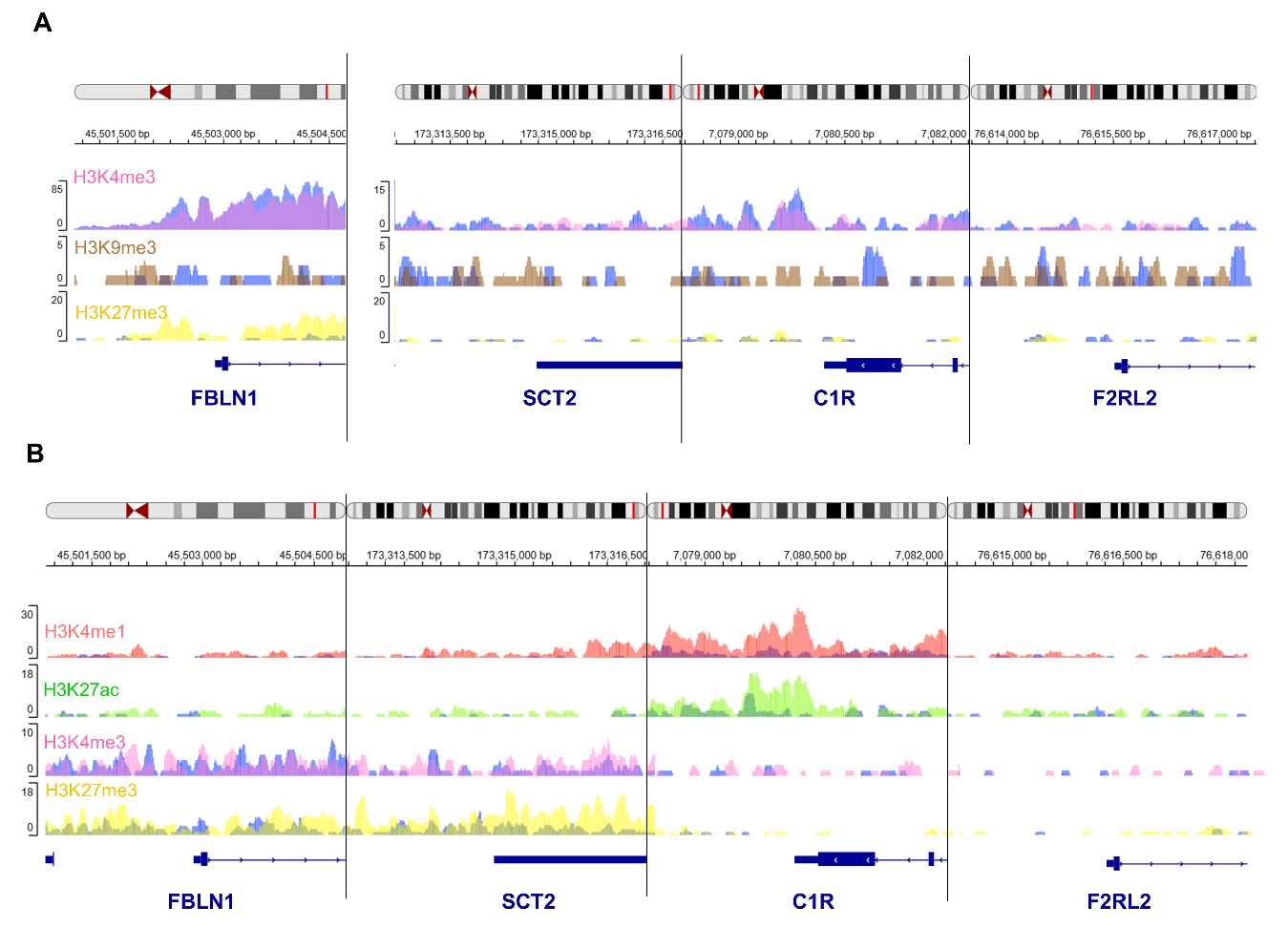


**Supplementary Figure 3.** Analysis of HPM of the prognostic Ca^2+^-signaling gene signature. Panel A displays the analysis using the GSE113879 dataset, which included adjacent liver tissue and HBV-HCC. The H3K4me3 mark associated with euchromatin is shown in lilac, while the H3K9me3 and H3K27me3 marks associated with heterochromatin are represented in brown and yellow, respectively. Panel B presents the analysis of the GSE112221 dataset, including normal liver and non-HBV HCC samples. Three marks associated with euchromatin, H3K4me1 (red), H3K27ac (green), and H3K4me3 (lilac), are contrasted with the repressive heterochromatin mark H3K27me3 (yellow). Blue peaks correspond to histone modifications in adjacent control liver samples. The x-axis represents the genomic position relative to the TSS (-2000 bp to +2000 bp), and the y-axis indicates the normalized intensity of the ChIP-seq signal. The blue arrows at the bottom indicate gene orientation.


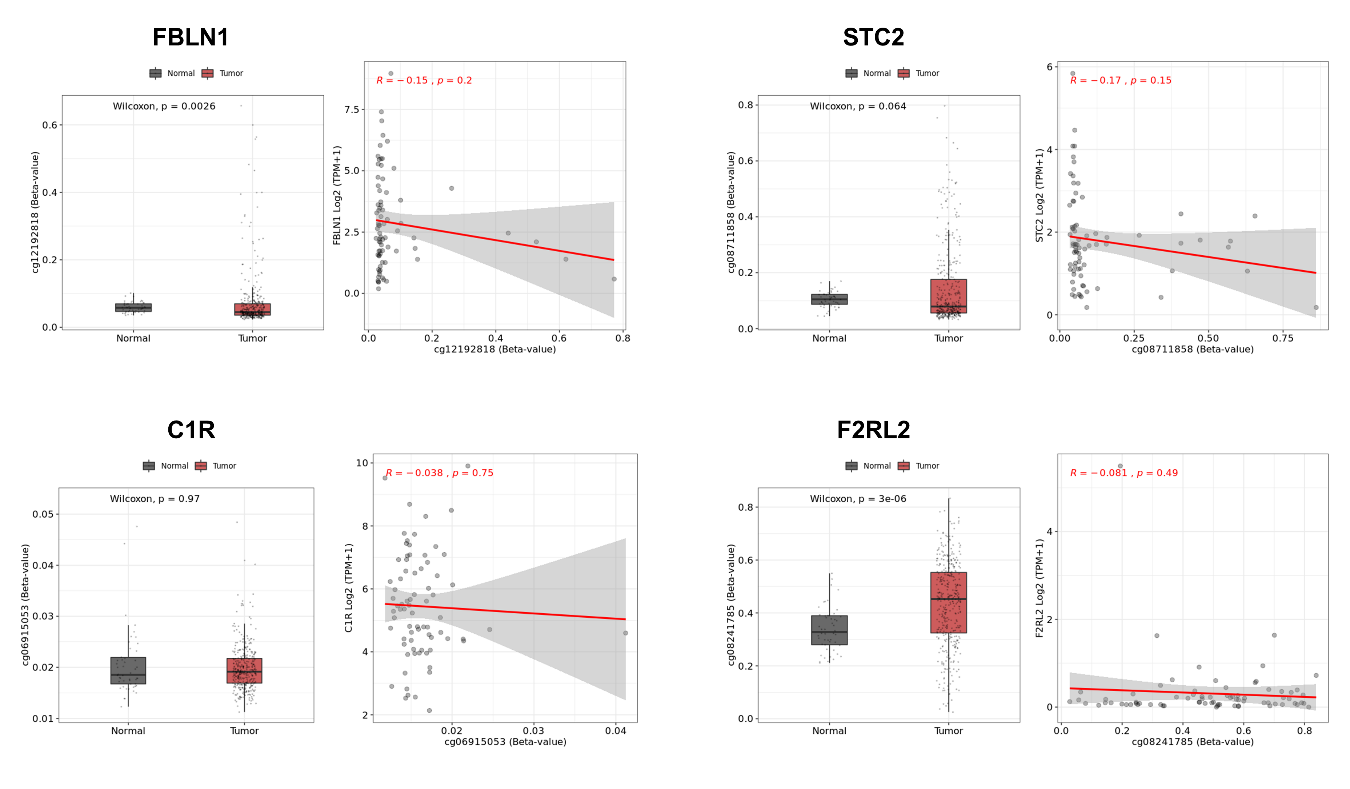


**Supplementary Figure 4.** Analysis of DNA methylation of the prognostic Ca^2+^-signaling gene signature. DNA methylation was measured using CpG probes located 200 bp upstream of the transcription start site. The y-axis indicates β values representing the proportion of methylation (0 = unmethylated, 1 = fully methylated), while the x-axis corresponds to gene promoters. Additionally, correlation plots of DNA methylation with each gene´s expression are presented, based on Pearson's correlation. The SMART database was used for the analysis.


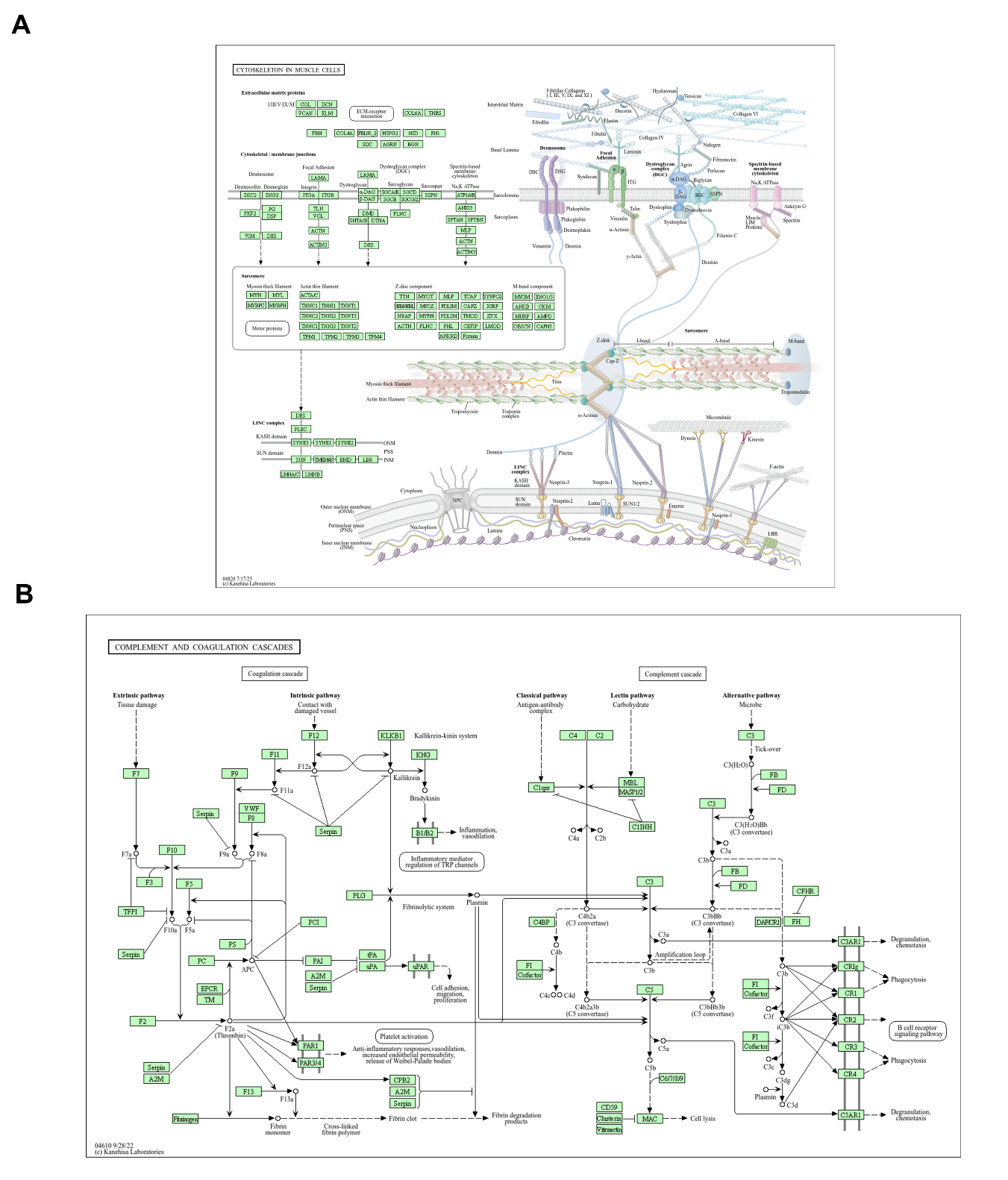


**Supplementary Figure 5.** Signaling pathways associated with Ca^2+^-regulated genes related to prognostic value. Panel A shows the association of *FBLN1* expression with cell adhesion and the cytoskeleton in muscle cells. Panel B shows the association between *C1R* and *F2RL2* expression and the complement and coagulation cascade pathways. The analysis was performed on the KEGG platform.
